# *Schisandra rubriflora* Fruit and Leaves as Promising New Materials of High Biological Potential: Lignan Profiling and Effect-Directed Analysis

**DOI:** 10.3390/molecules27072116

**Published:** 2022-03-25

**Authors:** Ewelina Sobstyl, Agnieszka Szopa, Michał Dziurka, Halina Ekiert, Hanna Nikolaichuk, Irena Maria Choma

**Affiliations:** 1 Department of Chromatography, Faculty of Chemistry, University of M. Curie-Skłodowska, M. Curie-Skłodowska Sq. 3, 20-031 Lublin, Poland; ewelina.sobstyl@gmail.com (E.S.); hanna.nikolaichuk@gmail.com (H.N.); 2 Chair and Department of Pharmaceutical Botany, Faculty of Pharmacy, Medical College, Jagiellonian University, Medyczna 9, 30-688 Krakow, Poland; halina.ekiert@uj.edu.pl; 3 Polish Academy of Sciences, The Franciszek Górski Institute of Plant Physiology, Niezapominajek Str. 21, 30-239 Cracow, Poland; m.dziurka@ifr-pan.edu.pl; 4 Department of Bioanalytics, Faculty of Biomedicine, Medical University of Lublin, Jaczewskiego Str. 8b, 20-090 Lublin, Poland

**Keywords:** dibenzocyclooctadiene lignans, TLC-DB, EDA, UHPLC–MS/MS, *Schisandra rubriflora*, red-flowered Chinese magnolia vine

## Abstract

The effect-directed detection (EDD) of *Schisandra rubriflora* fruit and leaves extracts was performed to assess their pharmacological properties. The EDD comprised TLC—direct bioautography against *Bacillus subtilis*, a DPPH assay, as well as α-glucosidase, lipase, tyrosinase, and acetylcholinesterase (AChE) inhibition assays. The leaf extracts showed stronger antioxidant activity than the fruit extract as well as inhibition of tyrosinase and lipase. The fruit extract was found to be extremely active against *B. subtilis* and to inhibit α-glucosidase and AChE slightly more than the leaf extracts. UHPLC–MS/MS analysis was carried out for the bioactive fractions and pointed to the possible anti-dementia properties of the dibenzocyclooctadiene lignans found in the upper TLC fractions. Gomisin N (518 mg/100 g DW), schisanhenol (454 mg/100 g DW), gomisin G (197 mg/100 g DW), schisandrin A (167 mg/100 g DW), and gomisin O (150 mg/100 g DW) were the quantitatively dominant compounds in the fruit extract. In total, twenty-one lignans were found in the bioactive fractions.

## 1. Introduction

Schisandraceae is a family of plants with three known genera: *Schisandra, Kadsura,* and *Illicium*. The family belongs to one of the oldest development lines for angiosperms—of the *Austrobaileyales* order. Especially well known is the *Schisandra* genus, which consists of about 30 species. *Schisandra* species are native to East and Southeast Asia [1,2,3].

The most known species is *Schisandra chinensis* Turcz. (Baill.)—Chinese magnolia vine. The raw material—fruit is well known in traditional Chinese medicine (TCM), all over the world [1,4,5]. Scientific studies confirmed the valuable traditional application of *Schisandrae chinensis fructus* (*Schisandra* fruit), the most important being hepatoprotective, anticancer, immunostimulant, and adaptogenic, as well as sedative and tonic effects [1,6]. In *S. chinensis* the most recognised active components responsible for *S. chinensis* bioactive properties are dibenzocyclooctadiene lignans, including, among others, schisandrin; gomisins A, C, and G; schisanhenol; deoxyschisandrin; and schisantherins A and B [1]. The dibenzocyclooctadiene lignans are a group of specific secondary metabolites, which are now considered also for neuroprotective and cognitive enhancement effects that can be used in neurological disorders [7,8].

*Schisandra rubriflora* (*S. rubriflora*) (Franch.) Rehd. et Wils is another interesting, but not much scientifically tested species of the *Schisandra* genus. *S. rubriflora* is less known because it is an endemic species. Its natural occurrence is the Western Sichuan province of China [2]. *S. rubriflora* cultivation in the field in Eastern Asia is difficult but possible. Sometimes *S. rubriflora* can be found in Europe as a plant grown for ornamental purposes [2,5,9]. The *S. rubriflora* fruits are known from TCM phytotherapy as a sedative and toning agent, so the species could be related to pharmacopoeial *S. chinensis.* This species is also traditionally used in the treatment of hepatitis, chronic gastroenteritis, and neurasthenia [2]. Up to now, the biological activity of fruit of this species has been described only by Chinese research groups and has been limited to the anti-HIV-1 studies, resulting from inhibition of HIV-1 replication in H9 lymphocytes. It was also proved that extracts from the shoots effectively reduce the level of GPT (glutamine-pyruvate transaminase) in blood, which may be useful in the treatment of liver and bile duct disorders [2]. Moreover, based on the studies of Szopa et al. [9], the anti-inflammatory effects of this species based on the inhibitory activity against 15-LOX, COX-1, and COX-2 enzyme assays have been indicated [9]. Additionally, studies confirmed the high antioxidant potential of *S. rubriflora* fruits, leaves, and stems based on FRAP, DPPH, and CUPRAC assays [10]. Generally, the phytochemical studies on *S. rubriflora* are rare. The lignans, especially dibenzocyclooctadiene lignans, as well as nortriterpenoids and bisnortriterpenoids are recognized as the main metabolites, but the differences in qualitative composition with *S. chinensis* are significant [8,10,11]. Recently, the lignan profiling of fruits, leaves, and stems of female and male *S. rubriflora* plants was elaborated [9]. Twenty-four lignans were confirmed with the UHPLC–MS/MS method; the main compounds in the extracts were schisanhenol; aneloylgomisin H and O; schisantherins A and B; schisandrin A; and gomisin O and G [9].

TLC is a widely used, simple, fast, and cost-effective tool for analyzing biological samples, including plant extracts. The method enables comparing many samples in one run, changing the separation conditions (various sorbents, eluents), methods of elution, and visualization. Nowadays, it offers a high-performance version (HPTLC), automation, and hyphenation with spectroscopic methods. TLC is also a preferable method in effect-directed analysis (EDA), which measures the effect emerging in a given biological system. Bioassays, which are the EDA tools, are performed directly on a TLC plate by so-called thin layer chromatography—direct bioautography (TLC-DB), which can be also named effect-directed detection (EDD). Detected biologically active compound can be further identified by spectroscopic methods such as MS, IR, and NMR [12,13,14,15,16,17]. TLC-DB can be based on any biological activity: antibacterial, antioxidant, estrogenic, or enzymatic. Recently, TLC-DB based on enzymatic inhibition has gained growing popularity. In the method, a TLC plate is immersed or sprayed with an enzyme solution and its substrate to obtain spots of enzyme inhibitors that differ in color from the background. The enzyme inhibitors may be the potential ingredients of drugs used to cure various diseases such as Alzheimer’s, Parkinson’s, depression, diabetes, obesity, and viral infections [18,19,20].

The acetylcholinesterase (AChE) enzyme activity assay is a popular enzyme test easily hyphenated with TLC. TLC-AChE inhibition detects AChE inhibitors (AChEI), which are currently the best form of pharmacotherapy of Alzheimer’s disease [21,22]. Another enzyme test used in TLC-DB is the glucosidase enzyme activity assay. α-Glucosidase inhibitors are responsible for lowering blood glucose levels. Drugs based on these compounds are used to fight diabetes [23]. The next is the lipase activity assay. Lipase enzyme inhibitors inhibit pancreatic lipase activity and, thus, reduce the amounts of fats absorbed into the body. These compounds are used in preparations to fight obesity and related diseases [24]. Tyrosinase inhibitors, detected in the tyrosinase activity assay, are responsible for the depigmentation effect—they inhibit melanin synthesis. These compounds are used in anti-atherosclerotic drugs or cosmetics to lighten discoloration [25,26].

Up to now, there have not been any scientific studies on the biological activities of *S. rubriflora* using TLC-EDA. Therefore, the aim of this research is the EDA of *S. rubriflora* extracts: fruit and leaves (taking into account the division of material originating from a male (M) and female (F) specimens) using various bioassays hyphenated with TLC. TLC-DB was used for searching for the antioxidants, antibacterial activity, as well as AChE, glucosidase, lipase, and tyrosinase inhibitors. Furthermore, the zones of AChE inhibition (TLC micro-preparative fractions) were subjected to LC-MS/MS to identify the most abundant lignans.

## 2. Results

### 2.1. TLC Screening Analysis of S. rubriflora Fruit and Leaves Extracts

**Figure 1:**
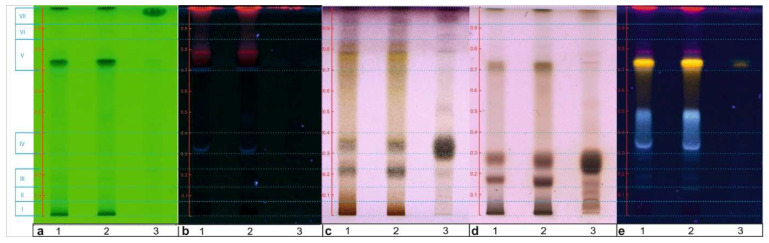
Chromatograms of *S. rubriflora* extracts: (1) *S. rubriflora* M leaves; (2) *S. rubriflora* F leaves; (3) *S. rubriflora* fruits; (**a**) 254 nm; (**b**) 366 nm; (**c**) AS (VIS); (**d**) thymol (VIS); (**e**) NP-PEG (366 nm). Mobile phase: F1; applied volume: 5 μL.

TLC linked to micro-chemical detection, i.e., derivatization (AS, thymol, NP-PEG), was used for fingerprinting the components of the *S. rubriflora* fruit and leaves extracts (Figure 1). At first, the AS assay was carried out. The AS is a universal reagent for detecting natural organic compounds (such as acids, phenols, sugars, lignans, steroids, and terpenes) (Figure 1c). The presence of saccharides in the extracts was confirmed by the thymol test (Figure 1d). In order to detect phenolic compounds, derivatization using the NP-PEG reagent was performed. The leaves extracts turned out to be rich in polyphenols, as evidenced by the fluorescent blue, yellow, and red spots (Figure 1e).

### 2.2. Effect-Directed Detection—TLC-DB

After the preliminary screening analysis (micro-chemical fingerprinting), we focused on searching for antioxidants, antibacterials, and enzymatic inhibitors in the extracts using TLC-DB, that is bioassays performed directly on TLC plates.

#### 2.2.1. DPPH Test

**Figure 2:**
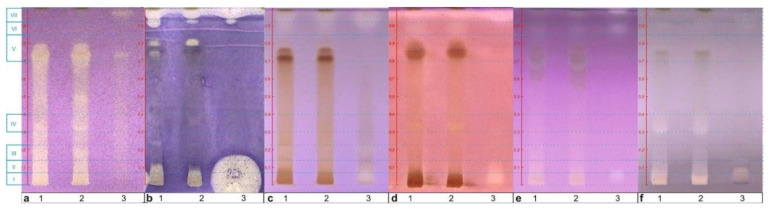
Bioautograms of the *S. rubriflora* extracts: (1) *S. rubriflora* M leaves; (2) *S. rubriflora* F leaves; (3) *S. rubriflora* fruits; (**a**) DPPH (VIS); (**b**) *B. subtilis* (VIS); (**c**) glucosidase inhibition (VIS); (**d**) AChE inhibition (VIS); (**e**) lipase inhibition (VIS); (**f**) tyrosinase inhibition (VIS). Mobile phase: F1; applied volume: 5 μL.

Testing for the antioxidant properties of the *S. rubriflora* fruit and leaves extracts was carried out using DPPH reagent sprayed onto the developed TLC plate. The DPPH test pointed to the presence of antioxidant compounds, in particular in the leaves of *S. rubriflora*. Compounds with antioxidant activity are visible as white-yellow spots on a purple background (Figure 2a, Section 4.3.3). It is worth paying attention to the bright spots in the leaves extract in the R_F_ range 0.3–0.8 and at R_F_ about 1, which coincide with the fluorescent zones in the NP-PEG test (Figure 1e). Thus, both tests confirm the presence of the antioxidant phenolic compounds. It should be noted that the TLC-DPPH test reveals also antioxidants other then polyphenols, as evidenced by the wide-range bright zone (R_F_ = 0–0.8).

#### 2.2.2. *Bacillus subtilis*

TLC-DB based on *B. subtilis* revealed the antibacterial properties of the tested species. The fruit extract is much more active against *B. subtilis* than the leaves ones (Figure 2b, Section 4.3.3). There is a large inhibition zone at the start, just as it was observed earlier in the case of the *S. chinensis* fruit where citric acid was detected [20].

#### 2.2.3. α-Glucosidase Inhibition Assay

The α-glucosidase inhibition assay confirmed the presence of glucosidase inhibitors in the fruit and leaves extracts (Figure 2c, Section 4.3.3). The fruits have slightly stronger inhibiting properties than the leaves. The bright zones visible against the light-purple background at R_F_ = 0.87–1 as well as at R_F_ = 0–0.10 and 0.18–0.22 prove the presence of the inhibitors in fruits. In the tracks of the leaves, “the lower” zones of α-glucosidase inhibition are partially masked by wide brown zones, probably related to polyphenols, while “the upper” zones are clearly visible at R_F_ = 0.90–1.

#### 2.2.4. AChE Inhibition Assay

The AChE inhibition test confirms the presence of AChE inhibitors in the investigated extracts (Figure 2d, Section 4.3.3), evidenced by bright spots on the intensely purple background. Compounds inhibiting the enzyme are found in the upper (fraction VI: R_F_ = 0.84–0.92 and fraction VII: R_F_ = 0.94–0.99 for leaves and fraction VII: R_F_ = 0.92–0.99 for fruit) and the lower (fraction I: R_F_ = 0–0.09 for fruits) parts of the plate. AChE inhibitors are also visible at the R_F_ = 0.31–0.36 (fraction IV) but only in the tracks related to the leaf extracts.

#### 2.2.5. Lipase Inhibition Assay

Based on the lipase inhibition assay, the enzyme inhibition properties were confirmed in the fruit and leaves extracts (Figure 2e, Section 4.3.3). Inhibition zones are found in the fruit and leaves extracts in the upper part of the track (fraction VI: R_F_ = 0.85–0.9, fraction VII: R_F_ = 0.99). They are especially evident in the fruit extract. Additionally, an inhibition zone of lipase is seen at the bottom of the TLC plate (fraction I: R_F_ = 0.03–0.08) in the fruit track. In the case of the leaf extracts, slightly visible zones at the R_F_ range 0.67–0.78 (fraction V) are partly masked by brown zones.

#### 2.2.6. Tyrosinase Inhibition Assay

Based on the test, it is possible to determine the presence of tyrosinase inhibitors—bright zones against the grey background (Figure 2f, Section 4.3.3). The leaves reveal stronger tyrosinase inhibitory properties than fruits. Besides the zones close to the start line (fraction I: R_F_ = 0–0.1), visible for all extracts, two additional zones are observed only for leaves at R_F_ = 0.3–0.4 (fraction IV) and at R_F_ = 0.78 (fraction V).

### 2.3. Micro-Preparative TLC

Micro-preparative analysis was performed for the methanol extracts of *S. rubriflora*. The obtained zones visible at 366 nm and 254 nm (fractions I–VII) (Figures S1 and S2) were eluted as described in Section 4.3.4 and subjected to UHPLC–MS/MS analysis.

### 2.4. UHPLC–MS/MS Lignan Targeted Profiling

The content of lignans found in the fractions obtained as a result of the TLC micro-preparative separation of the fruit as well as male (M) and female (F) leaf extracts of *S. rubriflora* was determined using UHPLC–MS/MS analysis based on external calibration for individual standards of lignans (Table S3). The results as well as the monitored fragmentation reactions and parameters of the analysis are summarized in Table 1 and Table 2 and Table S2, while the molecular formulas, weights, and chemical structures of the studied lignans are given in Table S1.

Twenty-one lignans, representing the five lignan groups dibenzocyclooctadiene lignans (schisantherin A and B, schisandrin, schisandrin C, gomisins A, D, G, J, N, O, 6-O-benzoylgomisin O, schisandrin A, rubrisandrin A, epigomisin O, schisanhenol, and rubriflorin A), aryltetralin lignan (wulignan A_1_), dibenzylbutane lignan (pregomisin), tetrahydrofuran lignan (fragransin A_2_), and dihydrobenzofuran neolignan (licarin A, B), were found and quantified.

The differences in the content of the individual compounds between the fruits and leaves (M, F) as well as among the analyzed fractions were observed.

The quantitatively dominant compound in the fruit extracts was gomisin N; its amount in all fractions was 518.44 mg/100 g DW (485.51 mg/100 g DW—fraction VII, 30.05 mg/100 g DW—fraction VI). High amounts were also confirmed for schisanhenol—totaling 454.34 mg/100 g DW (327.01 mg/100 g DW—fraction VII, 124.54 mg/100 g DW—fraction VI); gomisin G—197.18 mg/100 g DW (114.00 mg/100 g DW—fraction VII, 82.47 mg/100 g DW—fraction VI); schisandrin A—167.28 mg/100 g DW (143.14 mg/100 g DW—fraction VII, 23.41 mg/100 g DW—fraction VI); and gomisin O—149.67 mg/100 g DW (73.03 mg/100 g DW—fraction VII, 76.06 mg/100 g DW—fraction VI) (Table 1). The fruit extracts generally proved to be the richer source of lignans than leaves—totaling 1654.13 mg/100 g DW against 188.34 mg/100 g DW (M) and 324.30 mg/100 g DW (F) (Table 2).

Schisantherin A was found to be the most abundant compound in leaves—totaling 175.05 and 88.38 mg/100 g DW for F and M leaves, respectively, mostly in fraction VI (157.30 mg/100 g DW and 55.47 mg/100 g DW and for F and M leaves, respectively) (Table 1).

Two lignans (pregomisin and rubriflorin A) were found only as traces, independently of the fraction. The fragransin A_2_ and licarin A contents were also at a very low level (Table 1).

## 3. Discussion

Out of the analyzed fractions, the most abundant in lignans were fractions VII and VI (Table 2), totaling 84.98, 84.61, 1233.24 mg/100 g DW (fraction VII), and 86.58, 217.08, 409.08 mg/100 g DW (fraction VI) lignans in leaves M, leaves F, and fruits, respectively. On contrary, the fraction V has the lowest content of lignans, taking into account both the leaves and fruits (1.06, 2.50, 1.77 mg/100 g DW). The enormously high content of lignans in fraction VII is probably related to the zones of AChE inhibition seen at R_F_ 0.92–0.99, especially for fruits (Section 2.2.4 and Figure 2d). As it was proved, many dibenzocyclooctadiene lignans are responsible for AChE inhibition [20]. Hung et al. [27] carried out an AChE activity assay using an acetylthiocholine iodide substrate, based on a colorimetric method previously described by Ellman [28]. The high activity in the AChE assay was shown by schisantherin A, gomisin A, gomisin D, and gomisin G [27]. On the other hand, according to Hung et al. the lignans that should not inhibit AChE are schisandrin and gomisin N. The results of Hung et al. correlate with ours—the highest amounts of schisantherin A, gomisin A, gomisin D, and gomisin G are in fraction VII and VI (Figure 2d, Table 1). The dot-blot test showed that these lignans have AChE inhibitory activity (Figure S3, Table S4). In the dot-blot test, AChE activity was additionally shown by schisanhenol, schisandrin A, schisandrin C, and 6-O-beznoylgomisin O, which were also present in large amounts in the fruit extract. According to Hung et al., the inhibition of AChE is associated with methylenedioxy groups on the aromatic rings and the hydroxyl and benzoyl groups in the cyclooctadiene rings of lignans. Among the analyzed lignans (Table 1), schisantherin A and gomisin G have all mentioned the above groups, while gomisin A, gomisin D, gomisin O, schisantherin B, and epigomisin O have both methylenedioxy and hydroxyl groups. Fragransin A_2_ have only one hydroxyl group. Besides fragransin A_2_ (which is at traces in all fractions), gomisin O, epigomisin O, and schisantherin B were abundant in fractions VII and VI. Schisandrin C, showing high AChE activity in the dot-blot, has two methylenedioxy groups and no hydroxyl group. The structure of 6-O-benzoylogomisin contains both methylenedioxy and benzoyl groups.

The fraction VII for fruits contains a very large amount of gomisin N (485.51 mg/100 g DW), schisanhenol (327.01 mg/100 g DW), schisandrin A (143.11 mg/100 g DW), and gomisin G (114.00 mg/100 g DW) (Table 1). According to Hung et al., gomisin N does not inhibit AChE [27]. However, it showed weak AChE inhibitory activity in the dot-blot test (Figure S3). Besides, the structure contains one methylenedioxy group. Because the amount of gomisin N being very large, it can contribute to the inhibition zone related to fraction VII.

Fractions I and IV, which show inhibition zones in the AChE test (fraction I for fruits, fraction IV for leaves) (Figure 2d), have a rather low lignan content (fraction I: 1.36, 14.72, 1.40 mg/100 g DW in leaves M, leaves F, and fruits, respectively; fraction IV: 1.80, 1.30, 3.72 mg/100 g DW in leaves M, leaves F, and fruits, respectively), suggesting that their inhibiting activity against AChE is rather not related to lignans but to other, non-detected inhibitors.

Generally, all enzymatic inhibition assays pointed to the high activity of leaves. The same concerns the DPPH assay, and probably is related to the high content of polyphenols in leaves. According to the literature, glucosidase, AChE, lipase, and tyrosinase are inhibited by polyphenols [29,30,31,32]. It is commonly known that polyphenols have antioxidant properties [33].

This work presents for the first time such a detailed analysis of the individual lignan content (as many as 21 compounds) in fruits and leaves (both M and F) of *S. rubriflora*, taking into account their presence in various fractions obtained in TLC micro-preparative analysis. Qualitative studies on the *S. rubriflora* lignan composition were performed before only by Chinese teams [11,34,35,36,37]. Chen et al. [11] detected in fruit extracts schisandrin; schisantherin B; schisanhenol; deoxyschisandrin; angeloylgomisin P; tigloylgomisin P; gomisins M_1_, M_2_, O, and J; and rubrisandrins A and B—using the ^1^H NMR method. In 2010, Xiao et al. [37] identified in fruit extracts the following lignans with the use of ^1^D and ^2^D NMR spectroscopy: epiwulignan A_1_; gomisins G and O; angeloylgomisin P; wulignan A_2_; and rubrisandrin C. Mu et al. [36] made isolations from *S. rubriflora* fruit extracts and a further structural elucidation using preparative HPLC and ^13^C NMR of angeloygomisin Q; benzoylgomisin Q; schisandrin; schisandrins A and C; rubschizantherin; gomisins J, Q, C, B, K, N, S, and T; isogomisin O; wilsonilignangomisin G; and marlignans L and G. In extracts from aerial parts of *S. rubriflora*, Li et al. [35], using ^1^H and ^13^C NMR methods, identified gomisins K, M_1_, and R; dimethylgomisin J; interiotherin B; angeloylgomisin K_3_ and R; schisantherin D; mesodihydroguaiaretic acid; dihydroguaiaretic acid; and pregomisin. Li et al. [34] detected rubriflorin A and B in *S. rubriflora* stem extracts. According to our knowledge, for the first time, a small amount of licarin A was estimated in the material considered in our study.

## 4. Materials and Methods

### 4.1. Chemicals and Reagents

Acetic acid, ethanol 96%, ethyl acetate, methanol, thymol, sulfuric acid 95%, sodium acetate buffer, and phosphate buffer were from P.O.Ch. (Gliwice, Poland). Acetylcholinesterase enzyme (AChE) from *Electrophorus electricus*, *p*-anisaldehyde, bovine serum albumin (BSA), 1,1-diphenyl-2-picrylhydrazyl (DPPH), L-DOPA, Fast Blue B Salt, lipase from *Porcine pancreas*, 1-naphthyl acetate, 2-naphthyl acetate, 2-naphthyl α-D glucopyranoside, α-glucosidase from *Saccharomyces*, Natural Product—NP reagent (diphenylboryloxyethylamine), polyethylene glycol-4000, tyrosinase from mushroom and TRIS hydrochloride were purchased from Sigma Aldrich (Poznań, Poland). The MTT dye (3-(4,5-dimethyldiazol-2-yl)-2,5 diphenyltetrazolium bromide), HEPES buffer, and Triton X-100 were from Sigma Aldrich (Poznań, Poland). Agarose, Mueller-Hinton (M-H) agar, and M-H broth were purchased from Biocorp (Warsaw, Poland). The antibacterial activity was tested toward reference Gram-positive bacteria: *Bacillus subtilis* (ATCC 6633) was purchased from the American Type Culture Collections.

The standards of lignans were supplied by Chem-Faces Biochemical (Wuhan, China). The lignans working standards solutions were prepared in methanol.

### 4.2. Sample Origin and Preparation

Plant material was obtained in cooperation with Clematis—Źródło Dobrych Pnączy Sp. z o.o. (Pruszków, Poland) [38]. Plant species were identified by Dr (eng.) Szczepan Marczyński and Dr Agnieszka Szopa. For the experiments the fruits and leaves of about 10-year-old female (F) (100 individuals) and male (M) (50 individuals) *S. rubriflora* (Franch.) Rehd. et Wils specimens were collected in September 2017. The fruits were lyophilized (Labconco, Kansas City, MO, USA), and the leaves were air-dried (about 25–30 °C). Dry plant material was pulverized in a mixing ball mill (MM 400, Retch, Germany).

The *S. rubriflora* fruit and leaf extracts were obtained in methanol by maceration (72 h in a dark place at room temperature), using 0.5 g of DW (dry weight) per 5 mL. Then, the extracts were filtered through a filter paper and stored at −20 °C in dark bottles.

### 4.3. Methods

#### 4.3.1. Thin-Layer Chromatography

TLC was performed on 10 × 20 TLC Si60 F254 glass-backed plates (105715) from Merck (Darmstadt, Germany). The plant extracts were applied using the Linomat 5 automatic applicator from CAMAG (Muttenz, Switzerland). TLC plates were developed with the mobile phase: F1—ethyl acetate: methanol: water, 70:20:10 (*v*/*v*), using the DS horizontal sandwich chamber from Chromdes (Lublin, Poland). The mobile phase F1 was optimized to find the best separation of both polar and nonpolar components of *S.rubriflora* extract. Then, the plates were documented using TLC Visualiser 2 controlled via WinCATS software from CAMAG (Muttenz, Switzerland). Next, they were subjected to chemical derivatization or bioautography using a TLC automated spraying device or TLC sprayer from Merck (Darmstadt, Germany). A TLC Plate Heater from CAMAG (Muttenz, Switzerland) was used to heat the TLC plates after derivatization, when it was necessary.

#### 4.3.2. Chemical Derivatization

##### Anisaldehyde

For detection of phenols, steroids, and terpenes, an AS reagent was prepared. A total of 5 mL of sulfuric acid, 85 mL of methanol, 10 mL of acetic acid, and 0.5 mL of *p*-anisaldehyde were mixed. The developed plate was sprayed and heated for 10 min on a plate heater at T = 110 °C. The separated compounds were detected in visible light (VIS) as colored zones (violet, blue, red, grey, or green) [39].

##### Thymol

Sugar detection was performed with thymol. A total of 0.5 g of thymol was dissolved in 95 mL of ethanol and 5 mL of sulfuric acid was added. The developed plate was sprayed and heated for 15 min at T = 120 °C. The sugars were visible as brown zones in visible light (VIS) [39].

##### NP-PEG Reagent

Polyphenols detection was carried out with NP-PEG. The plate was sprayed with 1% NP reagent (diphenylboryloxyethylamine) and then a 5% ethanol solution of polyethylene glycol-4000 (PEG). Colored fluorescent spots were observed under 366 nm light [39].

#### 4.3.3. Effect-Directed Detection

##### TLC-DPPH

DPPH (α, α-diphenyl-β-picrylhydrazil) is a stable free radical with a dark purple color in solution and is used for the detection of antioxidants. The developed plate was sprayed with a 0.2% solution of DPPH in methanol. Yellow spots on the TLC plate on a purple background indicate the presence of an active antioxidant compound (VIS) [14].

##### TLC-DB—Bacillus subtilis

The developed TLC plate was immersed for 8 s in the bacterial suspension (8.0 × 10^7^ CFU/mL) using the TLC Immersion Device (CAMAG, Muttenz, Switzerland). Next, the plate was placed in a moistened plastic box and incubated at T = 37 °C for 17 h. Then, for visualization, the bioautogram was sprayed with a 0.2% MTT aqueous solution. To improve the intensity of the color, a drop of Triton X-100 was added per 10 mL of aqueous MTT solution. After reincubation at 37 °C for 0.5 h, white zones of bacterial growth inhibition were visible against a purple background. The bioautogram was digitized by the Visualizer [40].

##### TLC-DB—α-Glucosidase Inhibition

Inhibition of α-glucosidase was performed using the method described by Jamshidi-Aidji et al. [23]. The TLC plate was developed, dried, and sprayed with substrate solution (60 mg 2-naphthyl-*α*-D-glucopyranoside in 50 mL of ethanol) and allowed to dry. Then, the plate was sprayed with sodium acetate buffer (10.25 g of sodium acetate in 250 mL water, pH = 7.5 adjusted with acetic acid). The wet plate was sprayed with enzyme solution (100 units of *α*-glucosidase in 50 mL sodium acetate buffer). Next, the TLC plate was incubated for 10 min at T = 37 °C in a plastic box lined with wetted paper. For visualization, the plate was sprayed with a Fast Blue B Salt solution (100 mg in 100 mL of water). The *α*-glucosidase inhibitors were detected in visible light (VIS) as bright zones on the purple background.

##### TLC-DB—AChE Inhibition

Inhibition of AChE was performed directly on the TLC plate using the method described by Zhongduo et al. [22]. The TLC plate was developed, dried, and sprayed with the 0.05 M TRIS containing AChE (20 U) and BSA (150 mg) in 150 mL of the buffer. Then, the plate was sprayed with 2-naphthyl acetate dissolved in ethanol (25 mg per 100 mL). Next, the TLC plate was incubated for 0.5 h at T = 37 °C in a plastic box lined with wetted paper. After this time, the plate was sprayed with a Fast Blue B Salt solution (40 mg in 100 mL of water). The AChE inhibitors were detected in visible light (VIS) as bright spots on the purple background. Additionally, a dot-blot test was performed for the extracts and chosen standards (6-O-benzoylgomisin O, epigomisin O, gomisin A, gomisin D, gomisin G gomisin J, gomisin N, rubrisandrin A, schisandrin, schisandrin A, schisandrin C, schisanhenol, schisantherin A, schisantherin B, and wulignan A_1_).

##### TLC-DB—Lipase Inhibition

Lipase inhibition assay was performed using the method described by Hassan [24]. The TLC plate was developed, dried, and sprayed with substrate solution (150 mg 1-naphthyl acetate in 100 mL of ethanol) and allowed to dry. Next, the TLC plate was sprayed with the enzyme solution (50 mg lipase and 60 mg BSA dissolved in 47.5 mL water with 2.5 mL TRIS). Then, the TLC plate was incubated for 20 min at T = 37 °C in a plastic box lined with wetted paper. After this time, the plate was sprayed with a Fast Blue B Salt solution (50 mg in 100 mL of water). The lipase inhibitors were detected in visible light (VIS) as bright spots on the purple background.

##### TLC-DB—Tyrosinase Inhibition

Inhibition of tyrosinase was performed using the method described by Taibon et al. [25]. The TLC plate was developed, dried, and sprayed with substrate solution (120 mg L-Dopa in 49.5 mL 0.02 M in phosphate buffer with 0.5 mL Triton X-100). Then, the plate was sprayed with an enzyme solution (400 U tyrosinase in 1 mL of 0.02 M phosphate buffer). The plate was incubated in a plastic box at room temperature for 20 min. The tyrosinase inhibitors were detected in visible light (VIS) as bright spots on the gray background.

#### 4.3.4. TLC Micro-Preparative Analysis

The methanol extracts (50 μL) of *S. rubriflora* were applied as 5 cm band onto the analytical TLC plate (silica gel F_254_ 10 × 20 cm, 105715) and developed using the F1 mobile phase i.e., ethyl acetate: methanol: water (70:20:10 *v*/*v*/*v*). The separated fractions were scrapped, placed in the Eppendorf tube, and eluted with about 2000 μL of methanol. The eluates were concentrated by evaporating the solvent under a stream of nitrogen to 1700 μL. Then the fractions were subjected to UHPLC–MS/MS analysis.

#### 4.3.5. Lignan Profiling in Micro-Preparative Fractions

Targeted lignan profiling was carried out for the *S. rubriflora* micro-preparative fractions employing ultra-high-performance liquid chromatography coupled to a tandem mass spectrometer (UHPLC–MS/MS) using the modified method reported by Szopa et al. [9]. Samples were analyzed using a UHPLC Infinity 1260 system (Agilent, Waldbronn, Germany) coupled to a quadrupole tandem mass spectrometer 6410 MG/100 G DWQ LC/MS (Agilent, Santa Clara, CA, USA). Chromatographic separation was done on an analytical column (Kinetex C18 150 × 4.6 mm, 2.7 µm) in a gradient mode of 50% methanol in water (A) versus 100% methanol (B) with 0.1% formic acid in both phases. A linear gradient was applied—20% to 65% of B in 22 min at 0.5 mL/min at 60 °C—and the injection volume was 2 µL. The studied lignans and their structures are listed in Table S1. Lignans were analyzed in the MRM mode after positive ESI ionization (Table S2). For calibration, an external standard method was used. Data showing the method parameters, based on [41], are given in Table S3.

#### 4.3.6. Statistical Analysis

The differences in lignan content between leaves and fruit were evaluated by one-way ANOVA. Differences between the means were calculated using Duncan’s multiple range test (*p* < 0.05) using the statistical package STATISTICA 13.0 (Stat-Soft, Inc., Tulsa, OK, USA).

## Figures and Tables

**Table 1:** The individual lignan contents in the fractions of fruits as well as male (M) and female (F) leaves of *S. rubriflora*.

Lignans	Part of the Plant	Lignan Contents (mg/100 g DW)							
		Fractions							
		I	II	III	IV	V	VI	VII	Total Content in All Fractions
6-O-Benzoylgomisin O	Leaves (M)	0.02 ^b,c^	0.02 ^b^	0.02 ^b,c^	0.02 ^a^	nd *	0.12 ^c^	20.96 ^c^	21.18 ^c^
	Leaves (F)	0.24 ^a^	0.05 ^a,b^	0.05 ^b^	0.02 ^a^	0.02	4.18 ^a^	37.91 ^a^	42.48 ^a^
	Fruits	0.05 ^b^	0.02 ^b^	0.10 ^a^	0.02 ^a^	nd	1.43 ^b^	32.06 ^b^	33.68 ^b^
Epigomisin O	Leaves (M)	nd	0.02	nd	nd	nd	1.77 ^c^	0.85 ^b^	2.65 ^b,c^
	Leaves (F)	0.05 ^a^	nd	nd	nd	nd	5.08 ^b^	0.56 ^b^	5.68 ^b^
	Fruits	0.02 ^a,b^	nd	0.02	nd	nd	6.82 ^a^	6.22 ^a^	13.09 ^a^
Fragransin A_2_	Leaves (M)	0.02	tr **	tr	tr	tr	tr	tr	0.02 ^a,b^
	Leaves (F)	tr	tr	tr	0.05	tr	0.05	tr	0.10 ^a^
	Fruits	tr	tr	tr	tr	tr	tr	tr	tr
Gomisin A	Leaves (M)	nd	0.05	0.01 ^b^	0.01 ^b^	0.01 ^b^	2.15 ^b^	0.01 ^a,b^	2.25 ^b^
	Leaves (F)	nd	nd	nd	nd	nd	5.76 ^a^	0.05 ^a^	5.83 ^a^
	Fruits	0.02	nd	0.09 ^a^	1.6 ^a^	0.49 ^a^	1.85 ^b,c^	0.02 ^a,b^	2.52 ^b^
Gomisin D	Leaves (M)	0.05	tr	0.02 ^a^	0.05 ^a^	0.05 ^c^	14.04 ^a^	7.29 ^a^	21.49 ^a^
	Leaves (F)	tr	tr	tr	tr	0.12 ^b^	6.82 ^b^	4.01 ^b^	10.95 ^b^
	Fruits	tr	tr	0.02 ^a^	0.02 ^b^	0.39 ^a^	3.08 ^c^	4.49 ^b^	8.01 ^b,c^
Gomisin G	Leaves (M)	nd	nd	nd	nd	nd	0.12 ^b,c^	3.08 ^b^	3.21 ^b,c^
	Leaves (F)	1.43 ^a^	0.12 ^a^	0.15 ^b^	0.05 ^b^	0.07 ^a^	5.17 ^b^	3.30 ^b^	10.30 ^b^
	Fruits	0.10 ^b^	0.10 ^a^	0.34 ^a^	0.15 ^a^	0.02 ^b^	82.47 ^a^	114.00 ^a^	197.18 ^a^
Gomisin J	Leaves (M)	nd	0.1	nd	0.02 ^a^	0.02	0.17 ^b^	0.22 ^b^	0.53 ^c^
	Leaves (F)	0.07 ^a^	tr	nd	tr	tr	0.95 ^b^	0.36 ^b^	1.38 ^b^
	Fruits	0.02 ^b^	tr	0.02	0.02 ^a^	tr	2.60 ^a^	8.50 ^a^	11.17 ^a^
Gomisin N	Leaves (M)	0.13 ^b,c^	0.17 ^b^	0.07 ^c^	0.09 ^c^	0.04 ^c^	0.14 ^c^	8.10 ^b^	8.74 ^b,c^
	Leaves (F)	4.41 ^a^	0.58 ^a^	0.63 ^b^	0.29 ^b^	0.26 ^a^	1.13 ^b^	8.70 ^b^	16.02 ^b^
	Fruits	0.27 ^b^	0.42 ^a^	1.47 ^a^	0.57 ^a^	0.15 ^b^	30.05 ^a^	485.51 ^a^	518.44 ^a^
Gomisin O	Leaves (M)	nd	0.05 ^a^	nd	nd	nd	1.17 ^b^	2.89 ^b^	4.10 ^b,c^
	Leaves (F)	0.85 ^a^	0.07 ^a^	0.10 ^b^	0.05 ^b^	0.05 ^b^	9.74 ^b^	2.06 ^b^	12.92 ^b^
	Fruits	0.07 ^b^	0.05 ^a^	0.22 ^a^	0.10 ^a^	0.15 ^a^	76.06 ^a^	73.03 ^a^	149.67 ^a^
Licarin A	Leaves (M)	0.02	tr	tr	tr	0	0.17 ^a^	0.22 ^b^	0.41 ^a^
	Leaves (F)	nd	nd	nd	0.05	0.02	nd	0.32 ^a^	0.39 ^a^
	Fruits	tr	0.02	nd	tr	tr	0.05 ^b^	0.19 ^b^	0.27 ^b^
Licarin B	Leaves (M)	0.10 ^b^	1.34 ^a^	0.05 ^a^	0.15 ^a^	nd	0.07 ^b^	0.02 ^b^	1.72 ^b^
	Leaves (F)	0.17 ^a^	0.05 ^b^	0.05 ^a^	0.02 ^b^	0.07	0.10 ^b^	0.02 ^b^	0.49 ^c^
	Fruits	0.19 ^a^	tr	tr	tr	tr	1.63 ^a^	1.68 ^a^	3.50 ^a^
Pregomisin	Leaves (M)	tr	tr	tr	tr	tr	tr	tr	tr
	Leaves (F)	tr	tr	tr	tr	tr	tr	tr	tr
	Fruits	tr	tr	tr	tr	tr	tr	tr	tr
Rubriflorin A	Leaves (M)	tr	tr	tr	tr	tr	tr	tr	tr
	Leaves (F)	tr	tr	tr	tr	tr	tr	tr	tr
	Fruits	tr	tr	tr	tr	tr	tr	tr	tr
Rubrisandrin A	Leaves (M)	0.90 ^a^	9.08 ^a^	0.70 ^a^	1.31 ^a^	0.90 ^a^	1.34 ^a^	0.78 ^a^	15.01 ^a^
	Leaves (F)	0.10 ^b,c^	0.46 ^b^	0.17 ^b^	0.46 ^b^	0.85 ^a^	0.17 ^b^	0.22 ^b^	2.43 ^b^
	Fruits	0.15 ^b^	0.22 ^c^	0.78 ^a^	0.51 ^b^	0.32 ^b^	0.27 ^b^	0.80 ^a^	3.04 ^b^
Schisandrin	Leaves (M)	nd	0.05 ^a^	nd	0.02 ^a^	nd	3.25 ^b^	0.02 ^b^	3.35 ^b^
	Leaves (F)	nd	0.02 ^b^	0.02 ^a^	nd	0.12 ^a^	1.51 ^c^	0.05 ^a^	1.72 ^c^
	Fruits	0.02	0.02 ^b^	0.02 ^a^	0.02 ^a^	0.05 ^b^	6.75 ^a^	0.02 ^b^	6.92 ^a^
Schisandrin A	Leaves (M)	0.02 ^b,c^	0.05 ^b^	0.02 ^c^	0.02 ^c^	nd	0.22 ^b,c^	1.68 ^b^	2.02 ^b,c^
	Leaves (F)	1.46 ^a^	0.12 ^a^	0.19 ^b^	0.07 ^b^	0.07 ^a^	1.36 ^b^	2.50 ^b^	5.78 ^b^
	Fruits	0.07 ^b^	0.10 ^a^	0.39 ^a^	0.15 ^a^	0.02 ^b^	23.41 ^a^	143.14 ^a^	167.28 ^a^
Schisandrin C	Leaves (M)	0.05 ^b^	tr	nd	0.07	nd	tr	0.27 ^c^	0.39 ^b,c^
	Leaves (F)	0.10 ^a^	tr	tr	tr	0.58	tr	0.70 ^b^	1.38 ^b^
	Fruits	0.02 ^b,c^	0.02	0.07	tr	tr	0.24	10.56 ^a^	10.93 ^a^
Schisanhenol	Leaves (M)	0.02 ^c^	0.24 ^b,c^	0.02 ^c^	0.02 ^c^	0.02 ^c^	0.95 ^b^	1.46 ^b^	2.74 ^b^
	Leaves (F)	5.61 ^a^	0.49 ^a^	0.68 ^b^	0.22 ^b^	0.24 ^a^	1.58 ^b^	1.87 ^b^	10.69 ^b^
	Fruits	0.29 ^b^	0.32 ^b^	1.51 ^a^	0.53 ^a^	0.15 ^b^	124.54 ^a^	327.01 ^a^	454.34 ^a^
Schisantherin A	Leaves (M)	0.02 ^c^	0.32	0.05 ^a^	0.02 ^a^	0.02 ^a^	55.47 ^b^	32.47 ^a^	88.38 ^b^
	Leaves (F)	0.12 ^a^	nd	nd	nd	0.02 ^a^	157.30 ^a^	17.61 ^b^	175.05 ^a^
	Fruits	0.07 ^b^	nd	0.02 ^a,b^	0.02 ^a^	nd	13.99 ^c^	15.59 ^b,c^	29.70 ^c^
Schisantherin B	Leaves (M)	tr	0.01 ^a^	0.01 ^a^	tr	tr	5.41 ^b,c^	4.65 ^b^	10.09 ^c^
	Leaves (F)	0.10 ^a^	0.01 ^a^	tr	0.01 ^a^	0.01	16.15 ^a^	4.36 ^b^	20.63 ^a^
	Fruits	0.02 ^b^	0.01 ^a^	0.02 ^a^	0.01 ^a^	tr	6.54 ^b^	10.18 ^a^	16.79 ^b^
Wulignan A_1_	Leaves (M)	0.01 ^a^	0.02 ^a^	tr	tr	tr	0.02 ^b^	0.01 ^b^	0.06 ^b^
	Leaves (F)	0.01 ^a^	0.01 ^a^	0.01 ^a^	0.01	tr	0.03 ^b^	0.01 ^b^	0.08 ^b^
	Fruits	0.02 ^a^	tr	0.01 ^a^	tr	0.03	27.30 ^a^	0.24 ^a^	27.60 ^a^

Different letters (^a^, ^b^, ^c^) within each column for each compound indicate significant differences between the means (Duncan’s multiple range test; *p* < 0.05, *n* = 3). * nd—not detected with <LOD (limit of detection), detail given in Table S3. ** tr—traces with <LOQ (limit of quantification), detail given in Table S3.

**Table 2:** Total lignan content in the individual fractions of fruits as well as male (M) and female (F) leaves of *S. rubriflora*.

Part of the Plant	Lignan Contents (mg/100 g DW)							
	Fractions							
	I	II	III	IV	V	VI	VII	Total Content in All Fractions
Leaves (M)	1.36 ^b^	11.52 ^a^	0.97 ^c^	1.80 ^b^	1.06 ^c^	86.58 ^c^	84.98 ^b^	188.34 ^b,c^
Leaves (F)	14.72 ^a^	1.98 ^b^	2.05 ^b^	1.30 ^b,c^	2.50 ^a^	217.08 ^b^	84.61 ^b^	324.30 ^b^
Fruits	1.40 ^b^	1.30 ^b,c^	5.10 ^a^	3.72 ^a^	1.77 ^b^	409.08 ^a^	1233.24 ^a^	1654.13 ^a^

Different letters (^a^, ^b^, ^c^) within a column indicate significant differences between the means (Duncan’s multiple range test; *p* < 0.05, *n* = 3).

## Data Availability

Not applicable.

## Supplementary Materials

The following supporting information can be downloaded at: https://www.mdpi.com/article/10.3390/molecules27072116/s1, Figure S1.TLC micro-preparative analysis of *S. rubriflora* extracts (left: male leaves, right: fruits) (366 nm), Figure S2. TLC micro-preparative analysis of *S. rubriflora* extracts (left: male leaves, right: fruits) (254 nm), Figure S3. Dot-blot AChE assay of lignans standards (VIS). 1. 6-o-Benzoilgomisin O 2. Epigomisin O 3. Gomisin A 4. Gomisin D 5. Gomisin G 6. Gomisin J 7. Gomisin N 8. Rubrisandrin A 9. Schisandrin 10. Schisandrin A 11. Schisandrin C 12. Schisanhenol 13. Schisantherin A 14. Schisantherin B 15. Wulignan A, Table S1. The molecular formula, molecular weight and chemical structures of studied lignans, Table S2. The monitored fragmentation reactions (multiple reactions monitoring, MRM) for studied lignans. Ionization conditions applied: positive ionization (+ESI), drying gas temperature—350 °C, gas flow—12 L/min, nebulizer pressure—35 psi, Table S3. Calibration parameters for tested compounds, Table S4. Lignan contents vs their activities in AChE inhibition dot-blot test and information on their activities found in the literature.

## Sample Availability

Samples of the compounds are available from the authors.
