# Supplementary material for: Schisandra rubriflora Fruit and Leaves as Promising New Materials of High Biological Potential: Lignan Profiling and Effect-Directed Analysis
Source: Molecules. 2022 Mar 25;27(7):2116. doi: 10.3390/molecules27072116 (PMC9000568; doi:10.3390/molecules27072116)
Supplement: Supplementary file 1 [file molecules-27-02116-s001.zip › Supplememtary data molecules-1612968.pdf]

Schisandra rubriflora fruit and leaves as promising new materials of high biological potential: lignan profiling and effect-directed analysis

Ewelina Sobstyl[1], Agnieszka Szopa[2*], Michał Dziurka[3], Halina Ekiert[2], Hanna Nikolaichuk[1,4], Irena Maria Choma[1]**

[1] Department of Chromatography, Faculty of Chemistry, University of M. Curie-Skłodowska, M. Curie-Skłodowska Sq. 3, 20-031 Lublin, Poland; irena.choma@mail.umcs.pl (I.M.Ch.), ewelina.sobstyl@gmail.com (E.S.), hanna.nikolaichuk@gmail.com (H.N.)

[2] Chair and Department of Pharmaceutical Botany, Jagiellonian University, Medical College, Medyczna Str. 9, 30-688 Kraków, Poland; a.szopa@uj.edu.pl (A.S.), halina.ekiert@uj.edu.pl (H.E.)

[3] Polish Academy of Sciences, The Franciszek Górski Institute of Plant Physiology, Niezapominajek Str. 21, 30-239 Kraków, Poland; m.dziurka@ifr-pan.edu.pl (M.D.)

[4] Department of Bioanalytics, Faculty of Biomedicine, Medical University of Lublin, Jaczewskiego Str. 8b, 20-090, Lublin, Poland; hanna.nikolaichuk@gmail.com (H.N.)

Correspondence: *Agnieszka Szopa (A.S.): phone +48 12 620 54 36, fax +48 620 54 40, e-mail: a.szopa@uj.edu.pl, ORCID: 0000-0002-6351-4047; **Irena M. Choma (I.M.Ch.): phone +48 792 677 004, e-mail: irena.choma@mail.umcs.pl, ORCID: 0000-0002-6878-3748

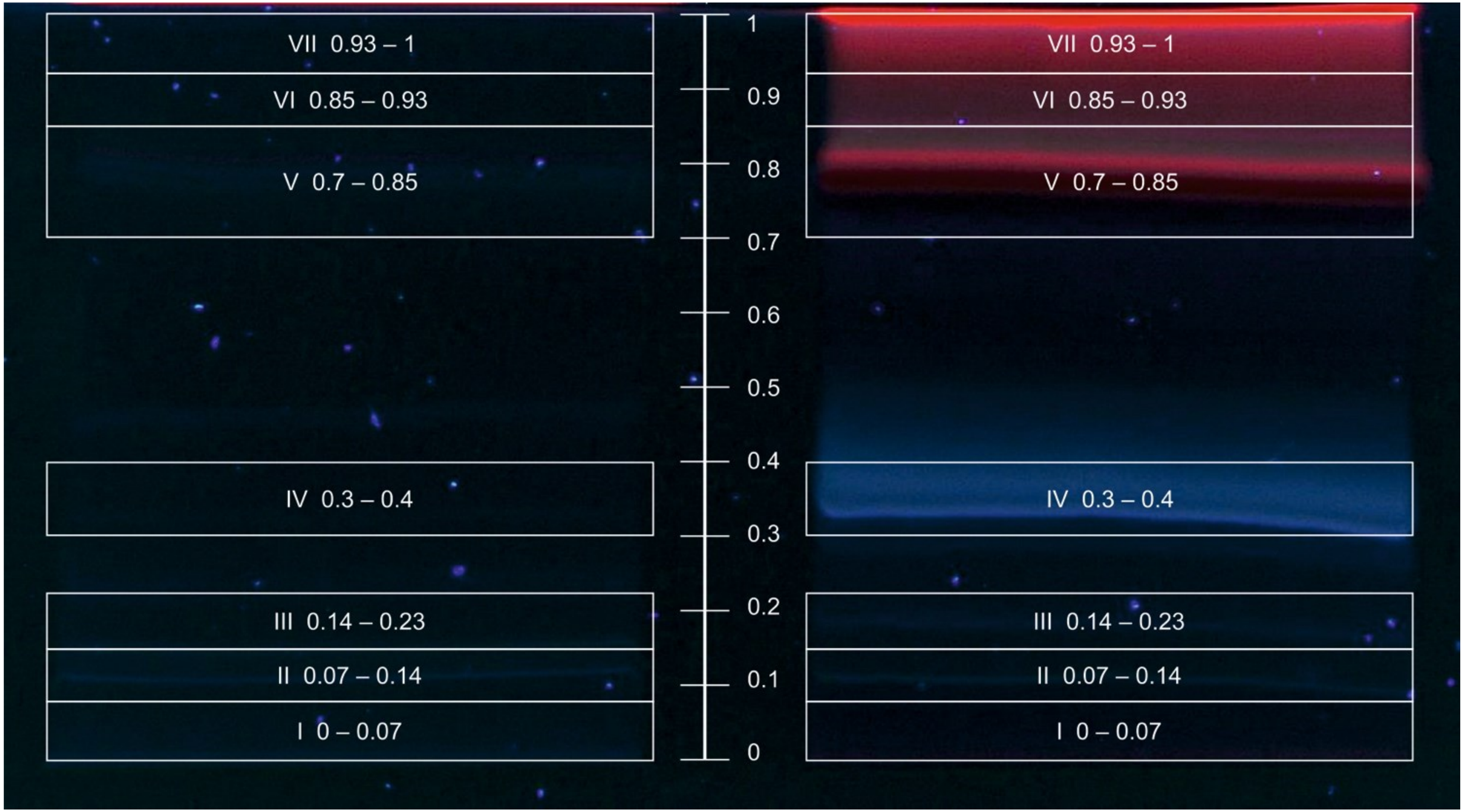

Figure S1.
TLC micro-preparative analysis of *S. rubriflora* extracts (left: male leaves, right: fruits) (366 nm).

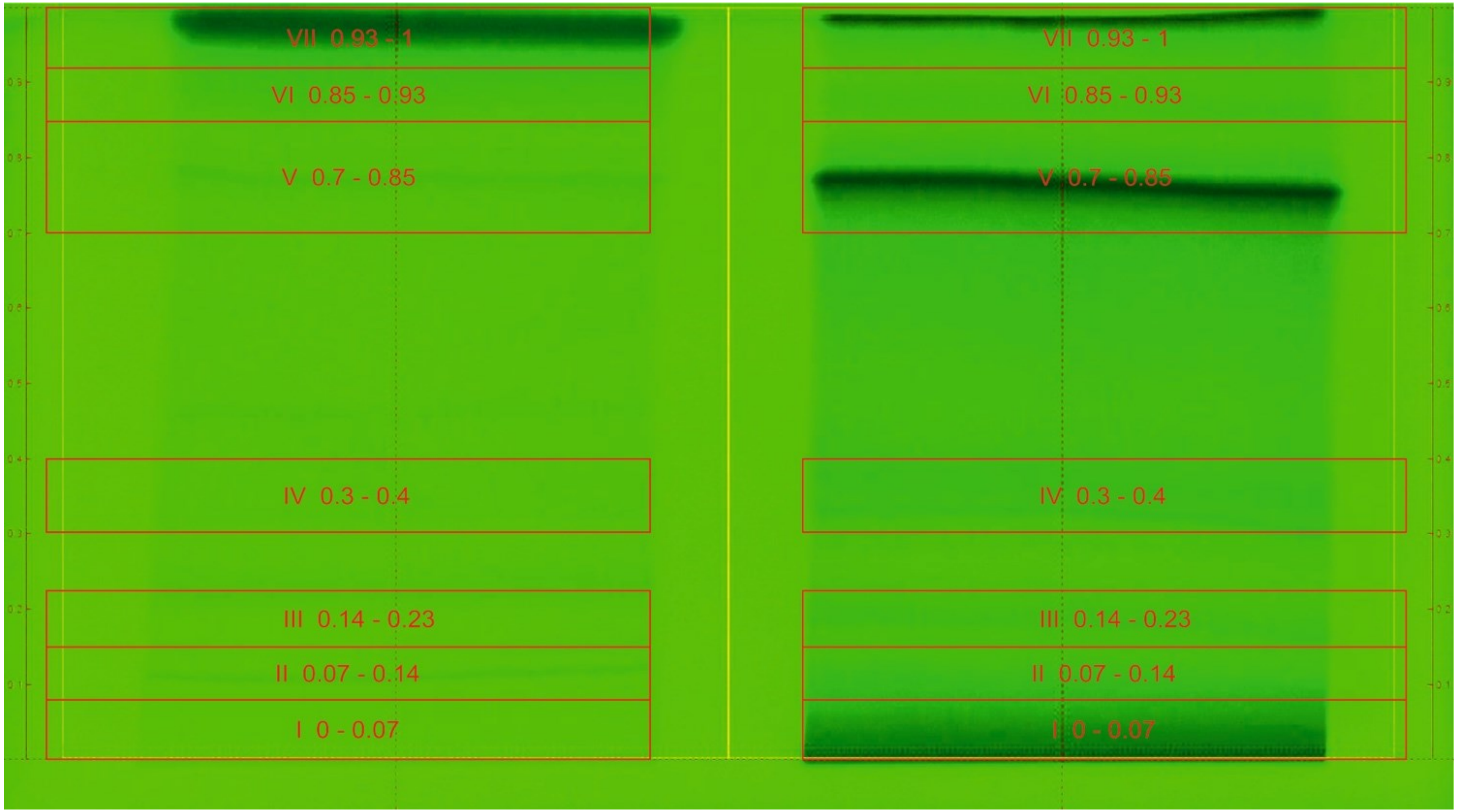

Figure S2.
TLC micro-preparative analysis of *S. rubriflora* extracts (left: male leaves, right: fruits) (254 nm).

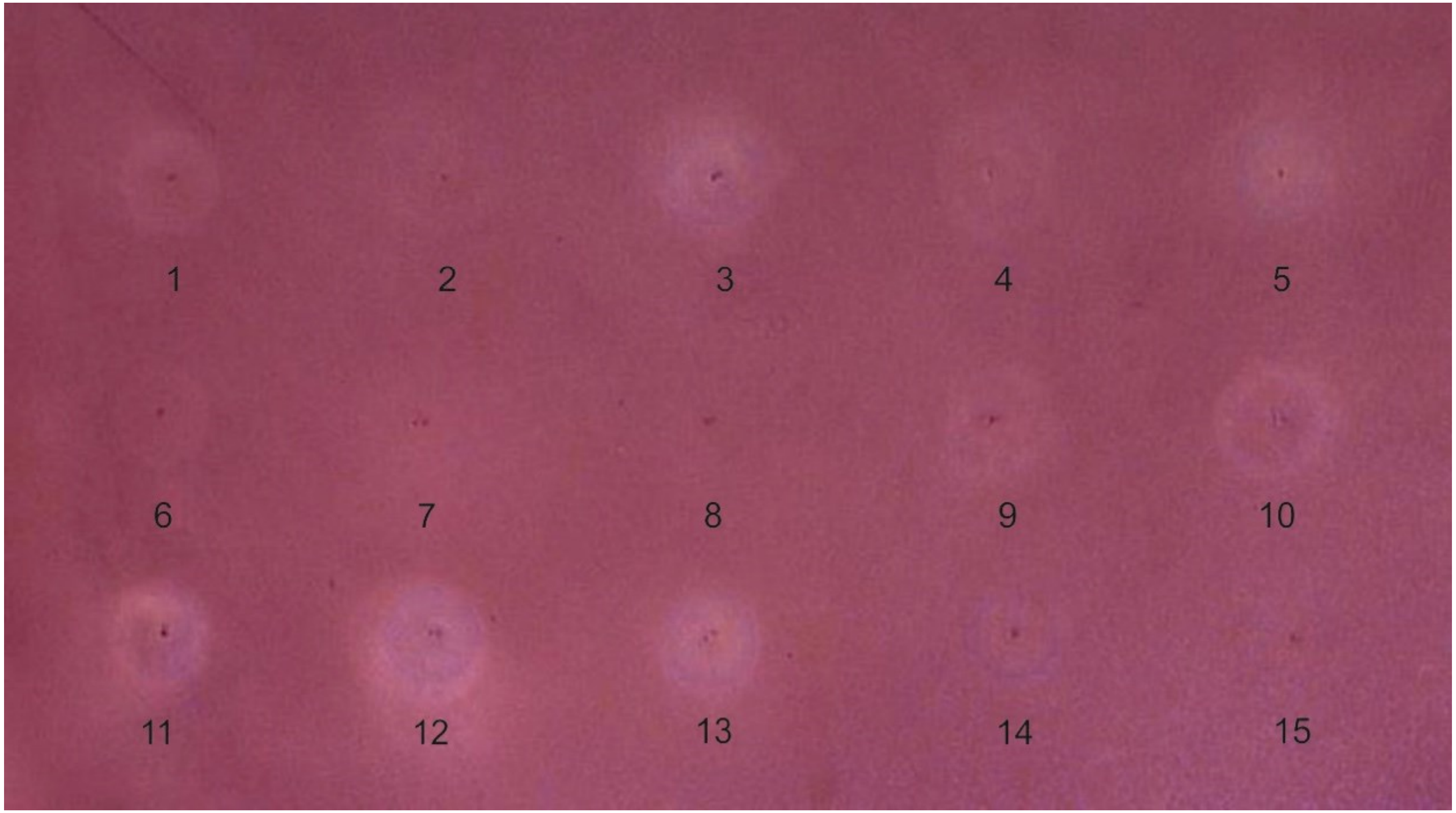

Figure S3.
Dot-blot AChE assay of lignans standards (VIS). 1. 6-o-Benzoilgomisin O 2. Epigomisin O 3. Gomisin A 4. Gomisin D 5. Gomisin G 6. Gomisin J 7. Gomisin N 8. Rubrisandrin A 9. Schisandrin 10. Schisandrin A 11. Schisandrin C 12. Schisanhenol 13. Schisantherin A 14. Schisantherin B 15. Wulignan A.

Table S1. The molecular formula, molecular weight and chemical structures of studied lignans

Lignan name	Molecular formula	Molecular weight	Chemical structure
6-O-Benzoylgomisin O	$C_{30}H_{32}O_8$	520.6	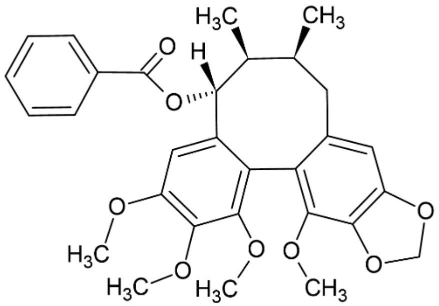
Epigomisin O	$C_{23}H_{28}O_7$	416.46	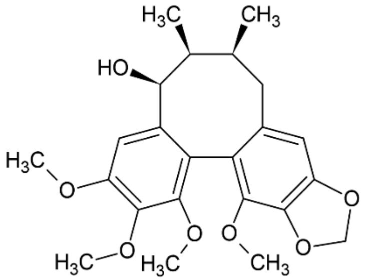

Fragransin A2	$C_{20}H_{24}O_5$	344.4	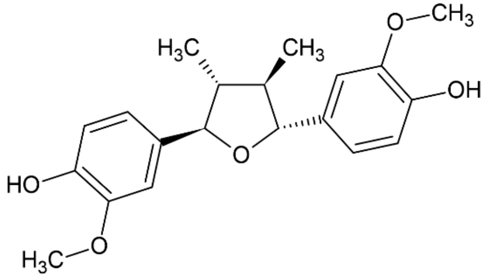
Gomisin A	$C_{23}H_{28}O_7$	416.5	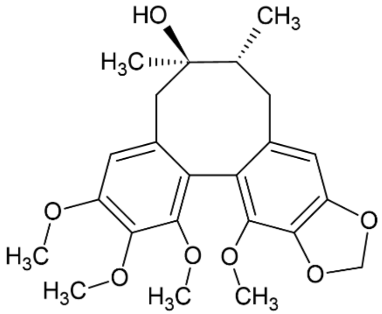
Gomisin D	$C_{28}H_{34}O_{10}$	530.56	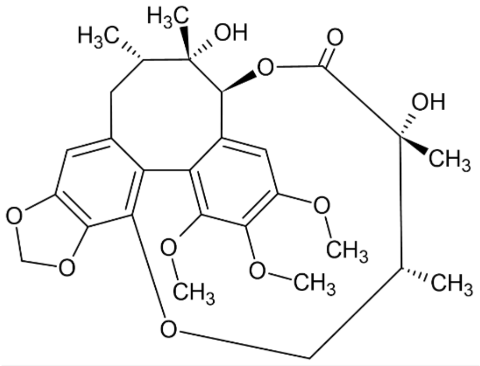

Gomisin G	$C_{30}H_{32}O_9$	536.6	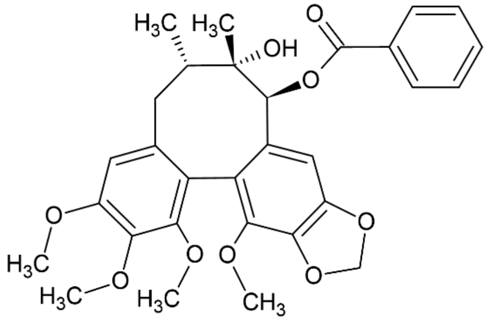
Gomisin J	$C_{22}H_{28}O_6$	388.19	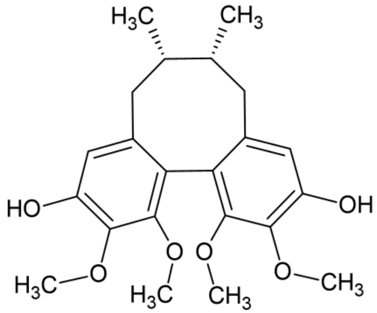
Gomisin N	$C_{23}H_{28}O_6$	400.46	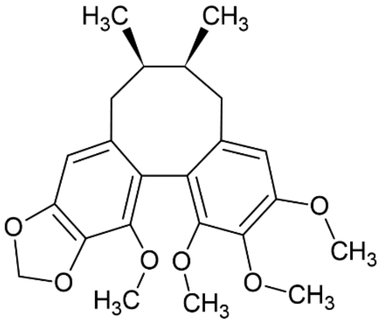

Gomisin O	$C_{23}H_{28}O_7$	416.46	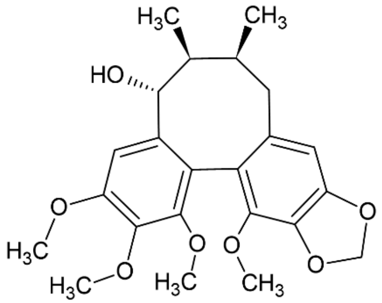
Licarin A	C20H22O4	326.4	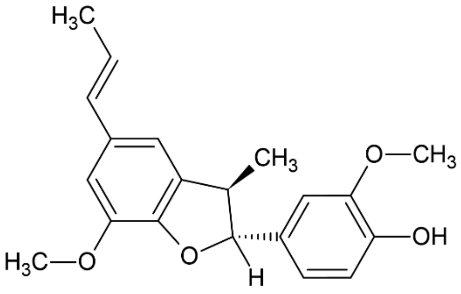
Licarin B	$C_{20}H_{20}O_4$	324.37	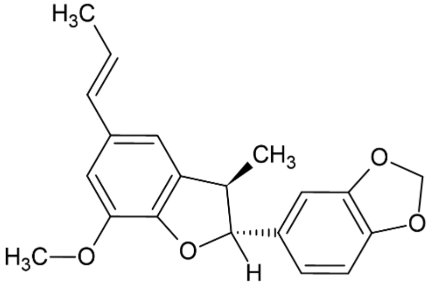

Pregomisin	$C_{22}H_{30}O_6$	390.47	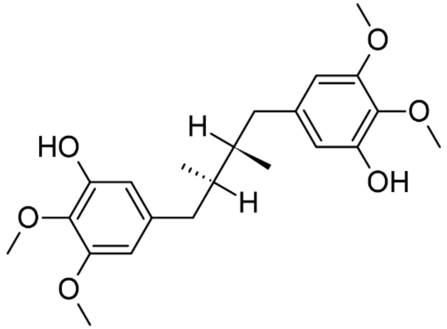
Rubriflorin A	$C_{30}H_{36}O_{10}$	556.6	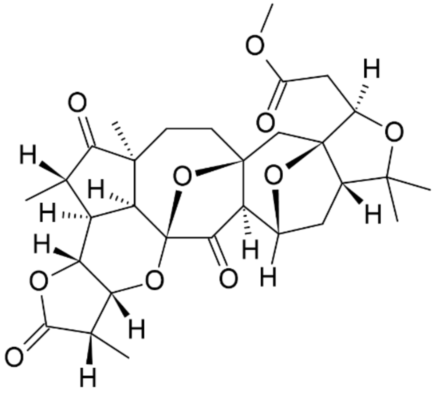
Rubrisandrin A	$C_{22}H_{28}O_6$	388.5	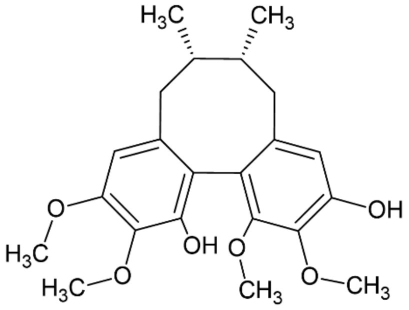

Schisandrin	$C_{24}H_{32}O_7$	432.5	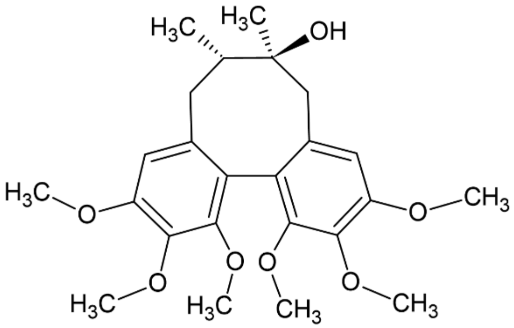
Schisandrin A	$C_{24}H_{32}O_6$	416.51	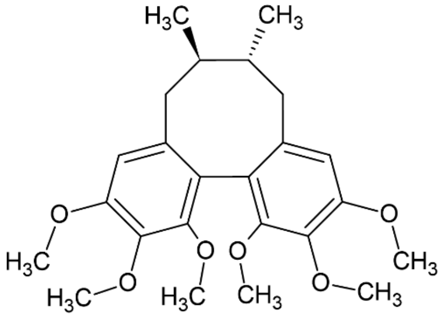
Schisandrin C	$C_{23}H_{24}O_6$	384.42	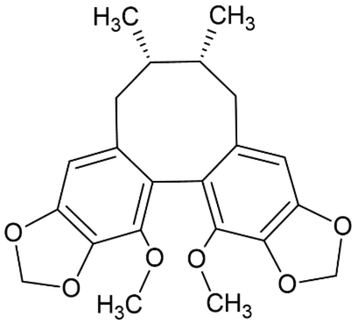

Schisanhenol	$C_{23}H_{30}O_6$	402.48	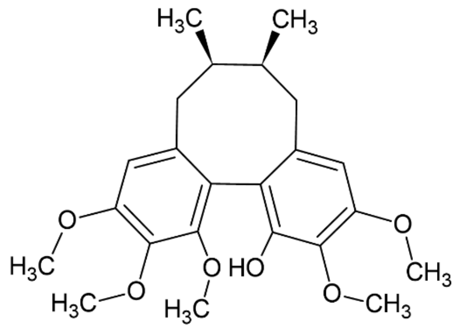
Schisantherin A	$C_{30}H_{32}O_9$	536.56	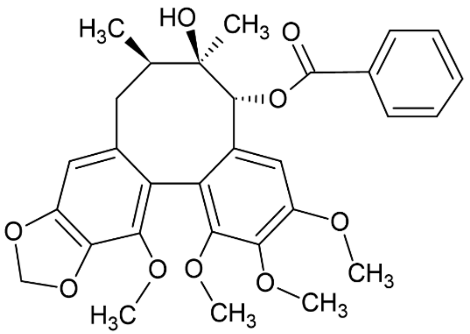
Schisantherin B	$C_{28}H_{34}O_9$	514.57	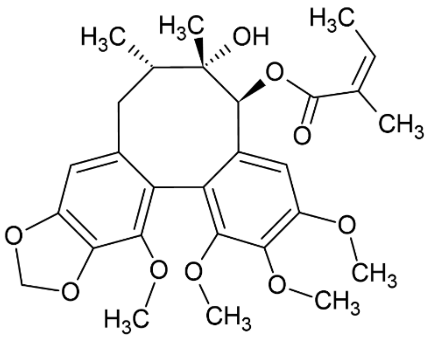

Wulignan A_1	$C_{20}H_{22}O_5$	342.4	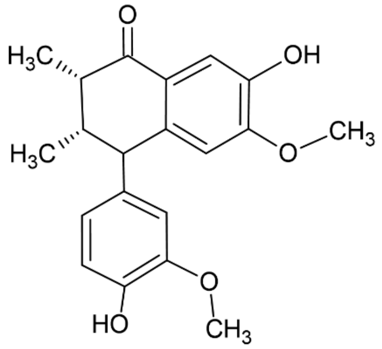

Table S2. The monitored fragmentation reactions (multiple reactions monitoring, MRM) for studied lignans. Ionization conditions applied: positive ionization (+ESI), drying gas temperature - 350°C, gas flow - 12 l/min, nebulizer pressure - 35 psi

Lignans	Monoisotopic Mass (Da)	Type of ion	Quantifier Transition	Fragmentor Voltage (V)	Collision energy (V)	Retention time ± SD (min)	MRM Time Segment
6-O-Benzoylgomisin O	520.2	[M-C7H6O2+H]+	399.3\368.2	100	150	22.22 ± 0,01	6
Epigomisin O	416.2	[M-H2O+H]+	399.3\368.2	100	150	11.95 ± 0,01	6
Fragransin A_2	344.2	[M-21]+	323.2\152.0	100	81	18.98 ± 0,01	12
Gomisin A	416.2	[M-H2O+H]+	399.3\368.2	100	150	10.03 ± 0,01	6
Gomisin D	530.2	[M-C6H10O3+H]+	401.3\168.0	150	77	8.52 ± 0,01	5
Gomisin G	536.2	[M-C7H6O2+H]+	415.3\371.1	150	13	10.99 ± 0,03	7
Gomisin J	388.2	[M+H]+	389.3\117.0	150	77	8.90 ± 0,01	5
Gomisin N	400.2	[M+H]+	401.3\168.0	150	77	20.81 ± 0,01	5
Gomisin O	416.2	[M-H2O+H]+	399.3\368.2	100	130	12.20 ± 0,01	8
Licarin A	326.2	[M+H]+	327.1/105.1	100	130	15.60 ± 0,01	10
Licarin B	324.1	[M+H]+	325.2\152.0	100	130	11.75 ± 0,04	8
Pregomisin	390.2	[M+H]+	391.3\139.0	150	61	19.71 ± 0,02	5
Rubriflorin A	556.2	[M+H]+	557.4\173.0	185	65	6.99 ± 0,03	4
Rubrisandrin A	388.2	[M-19]+	369.3\351.1	109	5	6.37 ± 0,02	3
Schisandrin	432.2	[M-H2O+H]+	415.3\359.2	150	13	7.97 ± 0,01	5
Schisandrin A	416.2	[M+H]+	417.3\316.2	160	21	17.97 ± 0,01	11
Schisandrin C	384.2	[M+H]+	385.3\231.1	130	17	22.70 ± 0,01	16
Schisanhenol	402.2	[M+H]+	403.3\231.0	150	130	14.98 ± 0,01	10
Schisantherin A	536.2	[M-C7H6O2+H]+	415.3\371.1	150	13	13.56 ± 0,01	7
Schisantherin B	514.2	[M-C5H8O2+H]+	415.3\371.1	150	13	13.97 ± 0,01	7
Wulignan A_1	342.1	[M+H]+	343.3\117.0	99	90	5.31 ± 0,01	2

Table S3. Calibration parameters for tested compounds

Lignans	Calibration curve equation (Linear model)	Coefficient of determination (R^2)	Tested linear range (ug/ml)	LOD (ug/ml)	LOQ (ug/ml)	Sample matrix	Spiked concentration (ug/ml)	Recovery (%)	RSD (%)
6-O-Benzoylgomisin O	y = 84527x + 617.85	0.9982	0.1~30	0.004	0.012	Leaves (M)	3.89	99.27	0.69
						Leaves (F)		100.45	0.56
						Fruits		102.13	1.57
Epigomisin O	y = 63954x - 141.21	0.999	0.1~30	0.005	0.016	Leaves (M)	3.41	99.99	0.01
						Leaves (F)		100.01	0.03
						Fruits		100.16	0.06
Fragransin A_2	y = 1515x - 87.184	0.9986	0.1~30	0.218	0.660	Leaves (M)	5.29	99.35	0.86
						Leaves (F)		98.81	0.96
						Fruits		99.03	0.89
Gomisin A	y = 78291x + 25645	0.9985	0.1~30	0.004	0.013	Leaves (M)	3.68	100.38	1.04
						Leaves (F		100.39	1.04
						Fruits		91.89	6.17
Gomisin D	y = 1089.3x - 289.56	0.9976	0.1~30	0.079	0.239	Leaves (M)	3.46	100.10	0.35
						Leaves (F)		100.29	0.25
						Fruits		92.62	4.04
Gomisin G	y = 40343x - 704.94	0.9974	0.1~30	0.008	0.025	Leaves (M)	3.46	100.06	0.12
						Leaves (F)		99.99	0.15
						Fruits		94.59	2.04
Gomisin J	y = 4192.4x - 245.16	0.9981	0.1~30	0.079	0.239	Leaves (M)	3.70	100.05	0.05
						Leaves (F)		100.04	0.05
						Fruits		89.56	5.32
Gomisin N	y = 17063x - 345.78	0.9993	0.1~30	0.019	0.059	Leaves (M)	3.81	105.05	5.46
						Leaves (F		105.05	5.46
						Fruits		86.17	8.15
Gomisin O	y = 76380x - 1167.7	0.9981	0.1~30	0.004	0.013	Leaves (M)	3.74	100.01	0.02
						Leaves (F)		100.06	0.06
						Fruits		94.94	4.99
Licarin A	y = 3485.2x - 273.51	0.9978	0.1~30	0.095	0.287	Leaves (M)	5.42	100.04	0.02

Lignans	Calibration curve equation (Linear model)	Coefficient of determination (R^2)	Tested linear range (ug/ml)	LOD (ug/ml)	LOQ (ug/ml)	Sample matrix	Spiked concentration (ug/ml)	Recovery (%)	RSD (%)
						Leaves (F)		100.03	0.02
						Fruits		99.99	0.01
Licarin B	y = 839.82x + 87.382	0.9986	0.1~30	0.039	0.119	Leaves (M)	1.92	100.40	0.19
						Leaves (F)		100.65	0.28
						Fruits		98.99	0.43
Pregomisin	y = 3993.4x - 138.18	0.9999	0.1~30	0.083	0.250	Leaves (M)	4.19	100.04	0.10
						Leaves (F)		100.03	0.10
						Fruits		100.06	0.13
Rubriflorin A	y = 14161x + 3460.4	0.9984	0.1~30	0.023	0.071	Leaves (M)	3.39	100.23	0.26
						Leaves (F)		100.23	0.28
						Fruits		99.47	0.62
Rubrisandrin A	y = 27485x + 346.46	0.9999	0.1~30	0.012	0.036	Leaves (M)	3.54	100.00	0.03
						Leaves (F)		100.01	0.03
						Fruits		99.98	0.02
Schisandrin	y = 17578x + 6671.3	0.9972	0.1~30	0.019	0.057	Leaves (M)	3.93	100.16	0.31
						Leaves (F)		100.16	0.31
						Fruits		90.92	5.98
Schisandrin A	y = 276081x - 832.02	0.9975	0.1~30	0.001	0.004	Leaves (M)	3.67	100.10	0.11
						Leaves (F)		100.10	0.11
						Fruits		97.54	7.54
Schisandrin C	y = 1342.4x - 24.449	0.9963	0.1~30	0.246	0.745	Leaves (M)	3.63	98.54	0.95
						Leaves (F)		98.70	0.99
						Fruits		99.73	0.69
Schisanhenol	y = 2469.4x - 272.74	0.9951	0.1~30	0.134	0.405	Leaves (M)	3.58	99.99	0.02
						Leaves (F)		100.00	0.03
						Fruits		92.08	7.86
Schisantherin A	y = 58357x - 1127.4	0.9993	0.1~30	0.06	0.17	Leaves (M)	3.47	100.32	0.27
						Leaves (F)		100.95	0.97
						Fruits		98.96	0.44
Schisantherin B	y = 29179x - 1127.4	0.9993	0.1~30	0.011	0.034	Leaves (M)	3.47	99.15	5.94

Lignans	Calibration curve equation (Linear model)	Coefficient of determination (R^2)	Tested linear range (ug/ml)	LOD (ug/ml)	LOQ (ug/ml)	Sample matrix	Spiked concentration (ug/ml)	Recovery (%)	RSD (%)
						Leaves (F)		102.35	7.45
						Fruits		89.72	1.13
Wulignan A_1	y = 5863x + 346.24	0.9993	0.1~30	0.056	0.171	Leaves (M)	3.90	99.99	0.05
						Leaves (F)		100.04	0.04
						Fruits		100.11	0.11

LOD – limit of detection 3.3×S/a; LOQ limit of quantitation 10×S/a; where S – standard deviation of blank sample, a- sensitivity (slope of calibration curve).
The recovery influenced by matrix effects was examined by comparing the MS signals (peak areas) for two sets of samples: lignans solutions in methanol and sample extracts spiked with lignans.
RSD relative standard deviation (n = 7 for leaves and n = 3 for fruits).

Table S4. Lignan contents vs their activities in AChE inhibition dot-blot test and information on their activities found in the literature

Lignans	Part of the plant	Lignan contents [mg/100g DW]		Dot-blot AChE	Reference [27]*
		VI	VII		
6-O-Benzoylgomisin O	Leaves (M)	0.12	20.96	++	
	Leaves (F)	4.18	37.91		
	Fruits	1.43	32.06		
Epigomisin O	Leaves (M)	1.77	0.85	+	
	Leaves (F)	5.08	0.56		
	Fruits	6.82	6.22		
Fragransin A_2	Leaves (M)	tr	tr		
	Leaves (F)	0.05	tr		
	Fruits	tr	tr		
Gomisin A	Leaves (M)	2.15	0.01	+++	+
	Leaves (F)	5.76	0.05		
	Fruits	1.85	0.02		
Gomisin D	Leaves (M)	14.04	7.29	++	+
	Leaves (F)	6.82	4.01		
	Fruits	3.08	4.49		
Gomisin G	Leaves (M)	0.12	3.08	+++	+
	Leaves (F)	5.17	3.30		
	Fruits	82.47	114.00		
Gomisin J	Leaves (M)	0.17	0.22	+	-
	Leaves (F)	0.95	0.36		
	Fruits	2.60	8.50		
Gomisin N	Leaves (M)	0.14	8.10	+	-
	Leaves (F)	1.13	8.70		
	Fruits	30.05	485.51		
Gomisin O	Leaves (M)	1.17	2.89		
	Leaves (F)	9.74	2.06		
	Fruits	76.06	73.03		

Licarin A	Leaves (M)	0.17	0.22		
	Leaves (F)	0	0.32		
	Fruits	0.05	0.19		
	Leaves (M)	0.07	0.02		
Licarin B	Leaves (F)	0.10	0.02		
	Fruits	1.63	1.68		
	Leaves (M)	tr	tr		
Pregomisin	Leaves (F)	tr	tr		
	Fruits	tr	tr		
	Leaves (M)	tr	tr		
Rubriflorin A	Leaves (F)	tr	tr		
	Fruits	tr	tr		
	Leaves (M)	1.34	0.78		
Rubrisandrin A	Leaves (F)	0.17	0.22	+	-
	Fruits	0.27	0.80		
	Leaves (M)	3.25	0.02		
Schisandrin	Leaves (F)	1.51	0.05	++	-
	Fruits	6.75	0.02		
	Leaves (M)	0.22	1.68		
Schisandrin A	Leaves (F)	1.36	2.50	++	-
	Fruits	23.41	143.14		
	Leaves (M)	tr	0.27		
Schisandrin C	Leaves (F)	tr	0.70	+++	
	Fruits	0.24	10.56		
	Leaves (M)	0.95	1.46		
Schisanhenol	Leaves (F)	1.58	1.87	+++	
	Fruits	124.54	327.01		
	Leaves (M)	55.47	32.47		
Schisantherin A	Leaves (F)	157.30	17.61	+++	+
	Fruits	13.99	15.59		
	Leaves (M)	5.41	4.65		
Schisantherin B	Leaves (F)	16.15	4.36	+	
	Fruits	6.54	10.18		
	Leaves (M)	0.02	0.01		
Wulignan A_1	Leaves (F)	0.03	0.01	+	
	Fruits	27.30	0.24		

+	low activity
++	high activity
+++	very high activity
-	no activity

* [27] - Hung, T.M.; Na, M.; Min, B.S.; Ngoc, T.M.; Lee, I.S.; Zhang, X.; Bae, K. Acetylcholinesterase inhibitory effect of lignans isolated from Schizandra chinensis. *Arch. Pharm. Res.* **2007**, *30*, 685–690, doi:10.1007/bf02977628.
